# Gaining and sustaining schistosomiasis control: study protocol and baseline data prior to different treatment strategies in five African countries

**DOI:** 10.1186/s12879-016-1575-2

**Published:** 2016-05-26

**Authors:** Amara E. Ezeamama, Chun-La He, Ye Shen, Xiao-Ping Yin, Sue C. Binder, Carl H. Campbell, Stephen Rathbun, Christopher C. Whalen, Eliézer K. N’Goran, Jürg Utzinger, Annette Olsen, Pascal Magnussen, Safari Kinung’hi, Alan Fenwick, Anna Phillips, Josefo Ferro, Diana M. S. Karanja, Pauline N. M. Mwinzi, Susan Montgomery, W. Evan Secor, Amina Hamidou, Amadou Garba, Charles H. King, Daniel G. Colley

**Affiliations:** Department of Epidemiology & Biostatistics, University of Georgia, Athens, GA USA; Center for Tropical and Emerging Global Diseases, University of Georgia, Athens, GA USA; Unité de Formation et de Recherche Biosciences, Université Félix Houphouët-Boigny, Abidjan, Côte d’Ivoire; Centre Suisse de Recherches Scientifiques en Côte d’Ivoire, Abidjan, Côte d’Ivoire; Swiss Tropical and Public Health Institute, Basel, Switzerland; University of Basel, Basel, Switzerland; University of Copenhagen, Copenhagen, Denmark; National Institute for Medical Research, Mwanza, Tanzania; Schistosomiasis Control Initiative, Imperial College, London, UK; Catholic University of Mozambique, Beira, Mozambique; Centre for Global Health Research, Kenya Medical Research Institute, Kisumu, Kenya; Centers for Disease Control and Prevention, Atlanta, GA USA; Réseau International Schistosomoses, Environnement, Aménagement et Lutte (RISEAL-Niger), Niamey, Niger; Center for Global Health and Diseases, Case Western Reserve University, Cleveland, OH USA; Department of Microbiology, University of Georgia, Athens, GA USA

**Keywords:** Schistosomiasis, *Schistosoma haematobium*, *Schistosoma mansoni*, Control, Preventive chemotherapy, Praziquantel, Côte d’Ivoire, Kenya, Mozambique, Niger, Tanzania

## Abstract

**Background:**

The Schistosomiasis Consortium for Operational Research and Evaluation (SCORE) was established in 2008 to answer strategic questions about schistosomiasis control. For programme managers, a high-priority question is: what are the most cost-effective strategies for delivering preventive chemotherapy (PCT) with praziquantel (PZQ)? This paper describes the process SCORE used to transform this question into a harmonized research protocol, the study design for answering this question, the village eligibility assessments and data resulting from the first year of the study.

**Methods:**

Beginning in 2009, SCORE held a series of meetings to specify empirical questions and design studies related to different schedules of PCT for schistosomiasis control in communities with high (gaining control studies) and moderate (sustaining control studies) prevalence of *Schistosoma* infection among school-aged children. Seven studies are currently being implemented in five African countries. During the first year, villages were screened for eligibility, and data were collected on prevalence and intensity of infection prior to randomisation and the implementation of different schemes of PZQ intervention strategies.

**Results:**

These studies of different treatment schedules with PZQ will provide the most comprehensive data thus far on the optimal frequency and continuity of PCT for schistosomiasis infection and morbidity control.

**Conclusions:**

We expect that the study outcomes will provide data for decision-making for country programme managers and a rich resource of information to the schistosomiasis research community.

**Trial registration:**

The trials are registered at International Standard Randomised Controlled Trial registry (identifiers: ISRCTN99401114, ISRCTN14849830, ISRCTN16755535, ISRCTN14117624, ISRCTN95819193 and ISRCTN32045736).

## Background

The Schistosomiasis Consortium for Operational Research and Evaluation (SCORE) was established through a grant provided to the University of Georgia (UGA) Research Foundation from the Bill & Melinda Gates Foundation in December 2008, to answer strategic questions about schistosomiasis control and elimination. SCORE’s focus is on operational research that will enhance the effectiveness of current and future schistosomiasis control programmes [1]. The SCORE portfolio includes evaluation of screening tests for *Schistosoma mansoni* [2, 3]; development of a gold-standard diagnostic test for *S. mansoni* and *Schistosoma haematobium* [4, 5]; assessment of the impact of drug pressure on parasite genetics [6]; research related to snail control [7]; the “rapid answers project” (RAP), which synthesises existing data to answer important programmatic questions; and studies on elimination of *S. mansoni* and *S. haematobium* in areas of very low prevalence [8, 9]. It also includes large field studies to compare multi-year strategies for preventive chemotherapy (PCT) in areas with moderate and high prevalence of schistosomiasis [1, 10]. This paper describes the decision-making process that informed the study design for these gaining and sustaining schistosomiasis control programmes that are facilitated by a series of cluster randomised trials with different PCT schemes. We present the results of eligibility assessments and year 1 data collection within 825 villages in five African countries where the studies are being conducted.

The prevalence of schistosome infection is usually highest in school-aged children (SAC) [11–15]. More than 90 % of schistosome infections occur in sub-Saharan Africa [16–18], resulting in at least 3.3 million disability-adjusted life years (DALYs) due to schistosomiasis-associated clinical and subtle morbidities [16, 19]. The World Health Organization (WHO) treatment guidelines call for PCT mainly targeting SAC in endemic areas through periodic administration of praziquantel (PZQ) [20], either through school-based treatment (SBT) or community-wide treatment (CWT) [1, 21–25]. The SCORE studies of gaining and sustaining control of schistosomiasis will compare the impacts and costs of different multi-year strategies involving CWT, SBT and “drug holidays” (i.e. years without PCT). Information from this research is expected to inform programme managers of national disease control programmes about the most effective strategies for gaining and sustaining the control of schistosomiasis and might also inform strategic shifts from morbidity control to interruption of transmission, therefore local elimination.

## Methods

### Process for designing SCORE’s operational research on gaining and sustaining control of schistosomiasis

In April 2009, SCORE convened a meeting involving researchers, programme managers and representatives of WHO to design the gaining and sustaining control studies. Discussions included the role of mapping, the need to integrate SCORE operational research with ongoing government programmes, integration of schistosomiasis control with other neglected tropical disease (NTD) prevention and control efforts (mainly soil-transmitted helminthiasis and lymphatic filariasis), data management for multi-country studies and concerns about ensuring the effectiveness and sustainability of PCT. It was decided that the studies would be large-scale cluster randomised trials in areas with starting prevalence of ≥10 % in SAC. Interventions would occur once a year, except in villages on “PZQ holiday” years, during which no drug administration will occur. Data would be collected annually, except in villages on “drug holiday” that year, starting before the first round of PCT and ending in the year after the fourth treatment round.

Based on this input, SCORE developed a request for proposals, which was disseminated to teams with a history of conducting relevant population-based helminthiasis research in Africa. Teams could propose to conduct studies of gaining control of *S. haematobium* (Sh2) or *S. mansoni* (Sm2), respectively, in areas with prevalence among SAC of ≥25 % or sustaining control (Sh1 or Sm1 studies, respectively) in areas with prevalence ranging between 10 and 24 %. Five study teams, each consisting of a principal investigator (PI) from the country where the study would take place and a northern partner, were selected to participate (Table 1). Criteria for selecting teams included likelihood that the proposed study areas would have sufficient numbers of villages meeting inclusion criteria, experience and track record of the team and proposed approach to ensuring high coverage of PCT.

**Table 1:** Study sites, study teams and numbers of villages screened in the eligibility surveys

Type of study	Country	Region of the country	Lead African partner institution	Lead Northern partner institution	# villages screened	# (%) villages that met criteria
Sustaining control	Côte d’Ivoire (Sm1)	Région des Montagnes and Région du Moyen Cavally	Université Félix Houphouët-Boigny; Abidjan, Côte d’Ivoire	Swiss Tropical and Public Health Institute; Basel, Switzerland	263	77 (29.3)
	Kenya (Sm1)	Kisumu region in western Kenya bordering Lake Victoria	Center for Global Health Research, Kenya Medical Research Institute (KEMRI); Nairobi, Kenya	Centers for Disease Control and Prevention (CDC); Atlanta, USA	150	75 (50.0)
	Niger (Sh1)	Dosso and Tillaberi regions in western Niger	Réseau International Schistosomoses, Environnement, Aménagement et Lutte (RISEAL-Niger), Niamey, Niger	Schistosomiasis Control Initiative (SCI), Imperial College London; London, UK	150	75 (50.0)
Gaining control	Kenya (Sm2)	Kisumu region in western Kenya bordering Lake Victoria	Center for Global Health Research, KEMRI; Nairobi, Kenya	CDC; Atlanta, USA	320	150 (46.9)
	Mozambique (Sh2)	Cabo Delgado province in northern Mozambique	Catholic University of Mozambique; Beira, Mozambique	SCI, Imperial College London; London, UK	150	150 (100.0)
	Niger (Sh2)	Dosso and Tillaberi regions in western Niger	National NTD Programme, Ministry of Health, Niamey, Niger	SCI, Imperial College London; London, UK	248	150 (60.5)
	Tanzania (Sm2)	Mwanza region bordering Lake Victoria	Mwanza Research Center, National Institute for Medical Research (NIMR); Mwanza, Tanzania	University of Copenhagen; Copenhagen, Denmark	308	167 (50.9)
Total					1,569	767 (48.3)

*Sh1* sustaining control study in *S. haematobium* moderate endemicity villages, *Sh2* gaining control study in *S. haematobium* high endemicity settings, *Sm1* sustaining control study in *S. mansoni* moderate endemicity villages, *Sm2* gaining control study in *S. mansoni* high endemicity villages

Mozambique and Niger were funded to conduct studies of *S. haematobium*, while Côte d’Ivoire, Kenya and Tanzania were funded to conduct studies of *S. mansoni.* Two teams (in Kenya and Niger) were funded to conduct studies of both gaining and sustaining control; in both countries gaining and sustaining studies occurred in separate areas. Côte d’Ivoire was funded to conduct a sustaining control study [26], and Mozambique and Tanzania were each funded to conduct gaining control studies (Table 1).

After being selected, the study teams participated in a “harmonization meeting” to review and refine the protocol. Protocols were standardised and plans were developed to ensure that data from comparable sites could be combined in subsequent analyses. In addition to finalising the study design and sample sizes, investigators agreed to a number of process measures and activities that were deemed essential for a successful study. These included robust plans for community sensitization, a commitment to measure coverage after each round of PCT and a willingness to revisit communities that did not achieve the coverage target to treat people who had been missed. Major discussion points and decisions from the initial and harmonization meetings are described in Table 2.

**Table 2:** Key decisions related to design of the gaining and sustaining control of schistosomiasis studies, and their rationale

Decision	Rationale
The study arms would not necessarily align with WHO recommendations for PCT	The WHO recommendations are not solidly evidence-based, and studying them would not answer most pressing questions
Sustaining schistosomiasis control studies would only involve SBT, and not adults or CWT	Existing data indicate adults are not major sources of transmission when prevalence of infection is <25 %, making testing and interventions for adults not cost-effective when resources are limited
Sustaining and gaining control of schistosomiasis studies would involve places with prevalence 10–24 % and ≥25 % in children aged 9–12 year, respectively	The cutoff of 10 % for sustaining studies was based on the idea that below that, one is moving towards elimination, and this will require additional interventions besides PCT. The choice of 25 % prevalence to divide gaining and sustaining studies was based on expert opinion
Sustaining control of schistosomiasis studies would include three arms, gaining studies would have six arms	SCORE would have preferred to test many more combinations of interventions, however this was not practical. The numbers of arms, numbers of villages per arm and number of children per village were an attempt to balance scientific, resource-related and practical considerations
Children aged 13–14 years would be tested to determine eligibility of a village for the sustaining or gaining control of schistosomiasis studies	Children who test positive must be treated. Testing children aged 9–12 years and treating those infected could affect the year 1 and subsequent study results, especially if prevalence is high. A very high prevalence could necessitate treating the entire village
“Drug holidays” would be included in study arms	The cost and impact of “drug holidays” is not known. If holidays have minimal negative effects on prevalence and intensity of *Schistosoma* infection in villages that have been targetted by PCT, holidays could allow for more widespread treatment
In all studies, first-year students would be tested at the beginning and end of the study	First-year students provide a measure of new infections in the community. If transmission is decreasing, prevalence and intensity in these children should fall
A convenience sample of adults would be tested in gaining control of schistosomiasis studies	Although initial plans called for a more systematic approach to identifying adults for testing, this proved impractical given the resources, so convenience samples were allowed
SCORE would provide mobile data collection software	Information provided at the harmonization meeting indicated that the software being used in lymphatic filariasis research could be readily adapted for SCORE use. This turned out not to be the case, but SCORE’s commitment to standardising data collection, providing support for data cleaning and storage and supporting mobile technology remained
SCORE-supported research needed to be conducted in close collaboration with Ministries of Health and Education	This was deemed essential both to ensure that PCT in SCORE study areas were conducted per protocol and to encourage the Ministries to use the results. In addition, it was assumed that PZQ access and use would work best when coordinated with the national schistosomiasis control programme
Study villages would need to achieve high levels of coverage; if these were not achieved during PCT, a team would need to return to the village to provide additional treatment	It was recognised that high coverage levels are not always achieved by PCT programmes. However, comparison of effectiveness among arms would require that treatments be delivered and actually consumed. Investigators were encouraged to have treatments directly observed to assure compliance
Investigators would be encouraged to publish their countries’ results; the SCORE secretariat would take responsibility for publishing combined results	In addition to encouraging widespread dissemination of the results of research, data sharing approaches that would allow investigators to use the data for modelling and other purposes were to be developed

*CWT* community-wide treatment, *PCT* preventive chemotherapy, *PZQ* praziquantel, *SBT* school-based treatment, *SCORE* schistosomiasis consortium for operational research and evaluation, *WHO* World Health Organization

### Study design

**Fig. 1:**
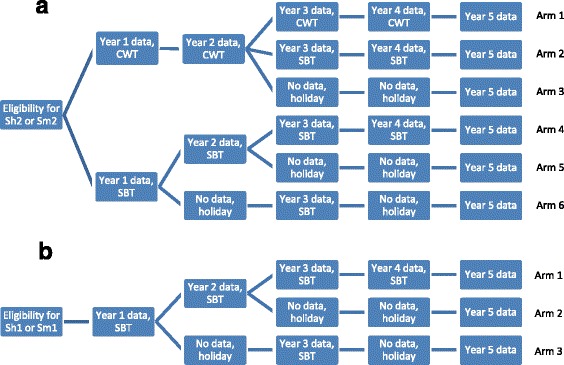
**a** Study arms and timeline for the studies of gaining control of schistosomiasis in Africa. CWT, community-wide treatment; SBT, school-based treatment; Sm2, gaining control study in *S. mansoni* endemic villages; Sh2, gaining control study in *S. haematobium* endemic villages. **b** Study arms and timeline for the studies of sustaining control of schistosomiasis in Africa. SBT, school-based treatment; Sh1, sustaining control study in *S. haematobium* endemic villages; Sm1, sustaining control study in *S. mansoni* endemic villages

Figure 1 shows the treatment arms for the gaining (*n* = 6 arms) and sustaining (*n* = 3 arms) control studies. The most intensive intervention arm in the gaining control studies involves four years of CWT, while the least intensive intervention arm involves two years of SBT alternating with “PZQ holiday” years. For sustaining control, the most intensive intervention arm involves four years of SBT, while the least intensive arms involve two years of SBT followed by two “PZQ holiday” years. The pattern of interventions in the three arms in the sustaining control studies matches that of arms 4, 5 and 6 in the gaining control studies.

A simple random allocation approach was used to assign all eligible villages to study arms except in Niger, where, in contradiction to the harmonized protocols, geographically clustered groups of 25 villages were randomly assigned to study arms.

The number of villages per arm and number of 9- to 12-year-old children tested per village were based on sample size calculations and practical considerations. Power computations were based on generalized estimating equations used to fit a logistic regression model that included treatment arm and time effects and treatment-by-time interaction, and assumed an over-dispersion parameter of φ = 5.0. Year 1 infection prevalence was assumed to be 50 % for gaining control studies and 25 % for sustaining studies. We assumed that the most intense treatment (arm 1) would reduce prevalence to a specific target level (15 and 10 % for gaining and sustaining control studies, respectively) by study end. We computed minimum effect sizes (difference between arm 1 and an alternative treatment arm at study conclusion) that could be detected with 90 % power for a two-sided α = 0.05 level test. Separate computations were carried out using various numbers of villages per treatment arm and children sampled per village. We assumed negligible correlation between measurements in year 1 and at study conclusion. Based on these assumptions, we estimated that 25 villages per treatment arm and 100 children aged 9–12 years per village were sufficient to detect absolute treatment differences of 11.4 and 9.4 % at the conclusion of the gaining and sustaining studies, respectively.

In addition to children aged 9–12 years, in the first and final fifth year, the protocol includes testing up to 100 children aged 5–8 years in both gaining and sustaining studies, and testing of 50 adults (aged 20–55 years) in the first and fifth years of the gaining studies. Populations to be tested in the different study years and methods of testing are shown in Table 3. No testing was to be conducted during “PZQ holiday” years because children found positive would need to be treated, and if the village prevalence was very high, it might be unethical to not treat the whole village.

**Table 3:** Populations tested in gaining and sustaining control of schistosomiasis studies, and microscopy performed, by year of the study

Intended population tested per village	Microscopy performed	Year 1	Years 2–4	Year 5
100 children aged 9–12 years	One mid-day urine specimen subjected to two filtrations; or three stool specimens subjected to duplicate Kato-Katz thick smears	Gaining and sustaining	Gaining and sustaining	Gaining and sustaining
100 (or as many as possible) children aged 5–8 years	One mid-day urine specimen subjected to two filtrations; or one stool specimen subjected to duplicate Kato-Katz thick smears	Gaining and sustaining		Gaining and sustaining
Adults	One mid-day urine specimen subjected to two filtrations; or one stool specimen subjected to duplicate Kato-Katz thick smears	Gaining		Gaining

### Eligibility surveys

To be eligible for inclusion in the five-year study, a village had to have a primary school (so that it could be randomised to SBT), at least 100 children aged 9–12 years and a starting prevalence of *Schistosoma* infection in the appropriate range (≥25 % for gaining and 10–24 % for sustaining control studies). Fifty children aged 13–14 years were tested in potentially eligible villages until the required number of villages (150 for gaining and 75 for sustaining control) were found. *S. mansoni* was assessed in these eligibility surveys by examining two slides from one stool sample per child using the Kato-Katz method [27], while *S. haematobium* was evaluated by microscopic examination of filtered urine or reagent strip testing for microhaematuria on a single mid-day urine specimen [13–15].

### Laboratory methods

For the eligibility surveys that involved children aged 13–14 years and for prevalence and intensity evaluations in adults and children 5–8 years of age, *S. mansoni* infection was based on microscopic examination of duplicate Kato-Katz thick smears from a single stool specimen [27]. For the annual cross-sectional prevalence and intensity assessment of *S. mansoni* in 9- to 12-year-old children, stool specimens were collected on three consecutive days from each child, and eggs enumerated on duplicate Kato-Katz thick smears per specimen.

The eligibility surveys assessed *S. haematobium* infection by either urine filtration microscopy [28, 29] or a reagent strip assay for microhaematuria [30] on a single urine specimen. For the cross-sectional prevalence and intensity surveys, two 10-ml aliquots from a single mid-day urine specimen were filtered, and the filters examined quantitatively under a microscope by two independent experienced laboratory technicians for *S. haematobium* eggs.

### Data collection and management

SCORE provided a list of variables to be collected on individual participants, as well as standard operating procedures (SOPs) for diagnosing schistosomiasis. Forms for collecting village-level data on such factors as water and sanitation, occupations and other contextual factors that might affect study results were also provided.

Most year 1 data were collected on paper. Kenya, Mozambique and Tanzania used a mobile-based system (EpiCollect®) developed at Imperial College London for data collection. Côte d’Ivoire used a mobile-based system (LINKS®) developed at the Task Force for Global Health for some data collection beginning in year 2. These systems involve data entry on-site, synching of data to a central server and downloading of data for cleaning and analysis.

### Statistical analysis

Simple univariate and bivariate analyses were used to describe study participant demographics. Differences in infection prevalence and intensity by study arm were assessed using logistic and Poisson regression models, respectively, with adjustment for over-dispersion. Calculations of infection intensity included only those individuals who had at least one positive slide; for those, intensity was the geometric mean on all their slides, including those without *Schistosoma* eggs detected.

### Ethics statement

Written informed consent was obtained from adults (including parents/legal guardians of children in the study) and assent was obtained from children less than 18 years old, except in places where village-level consent is the standard, in which case local requirements were met. Ethical review of research protocol was implemented by human subjects committee in each African country and by the institutional review board (IRB) of their respective northern partners. Sm1 and Sm2 studies in Kenya were reviewed and approved by the National Ethics Review Committee of the Kenyan Medical Research Institute (KEMRI; approval numbers SCC 1800 and SCC 1820, respectively) and by the IRB of the Centers for Disease Control and Prevention (CDC; approval #: 1661). For the Sm1 study in Côte d’Ivoire, ethical approval was obtained from the ethics committees in Côte d’Ivoire (reference no. 1994MSHP/CNER) and Basel (reference no. EKBB 279/10). In Niger, ethical approval was obtained from the Niger Republic National Consulate for ethical review (reference no. 012/2010/CCNE) and from the Imperial College Research Ethic Committee (ICREC_8_2_2). In Mozambique, ethical approval was received from the Ministry of Health (reference no. 235/CNBS/10) and the Imperial College Research Ethic Committee (ICREC_10_8_2). In Tanzania, ethical approval was obtained from the National Institute for Medical Research (NIMR; reference no. NIMR/HQ/R.8a/Vol. IX/1022). In addition to these, the UGA IRB implemented an administrative human subjects review and issued additional approval per country’s protocol as follows: 10021–0, 10221–0, 10267–0, 10353–0, 10431–0 and 10533–0 for Côte d’Ivoire, Kenya Sm1, Kenya Sm2, Tanzania, Niger and Mozambique, respectively.

The trials have been registered with the International Standard Randomised Controlled Trial registry under ISRCT numbers 99401114 (Côte d’Ivoire), 14849830 (Kenya Sm1), 16755535 (Kenya Sm2), 95819193 (Tanzania), 32045736 (Niger), and 14117624 (Mozambique).

## Results

### Eligibility surveys

Investigators in the gaining control studies (Fig. 1a) had to test between 150 and 320 villages to identify the 150 eligible villages; for sustaining studies, between 150 and 263 villages were surveyed to find 75 eligible villages (Table 1). The most common reason for excluding villages was that the prevalence among 13- to 14-year-old children was out of range. In addition, 20 villages in the Tanzania gaining studies were excluded because they had high rates (>10 %) of mixed *S. haematobium* and *S. mansoni* infections. Based on the added complexity of analysis and significant increases in funding required for testing to include areas with predominantly mixed infections, such settings were considered beyond the scope of the current SCORE study design.

### Year 1 surveys

#### Studies of gaining control (Kenya, Tanzania, Mozambique and Niger)

**Fig. 2:**
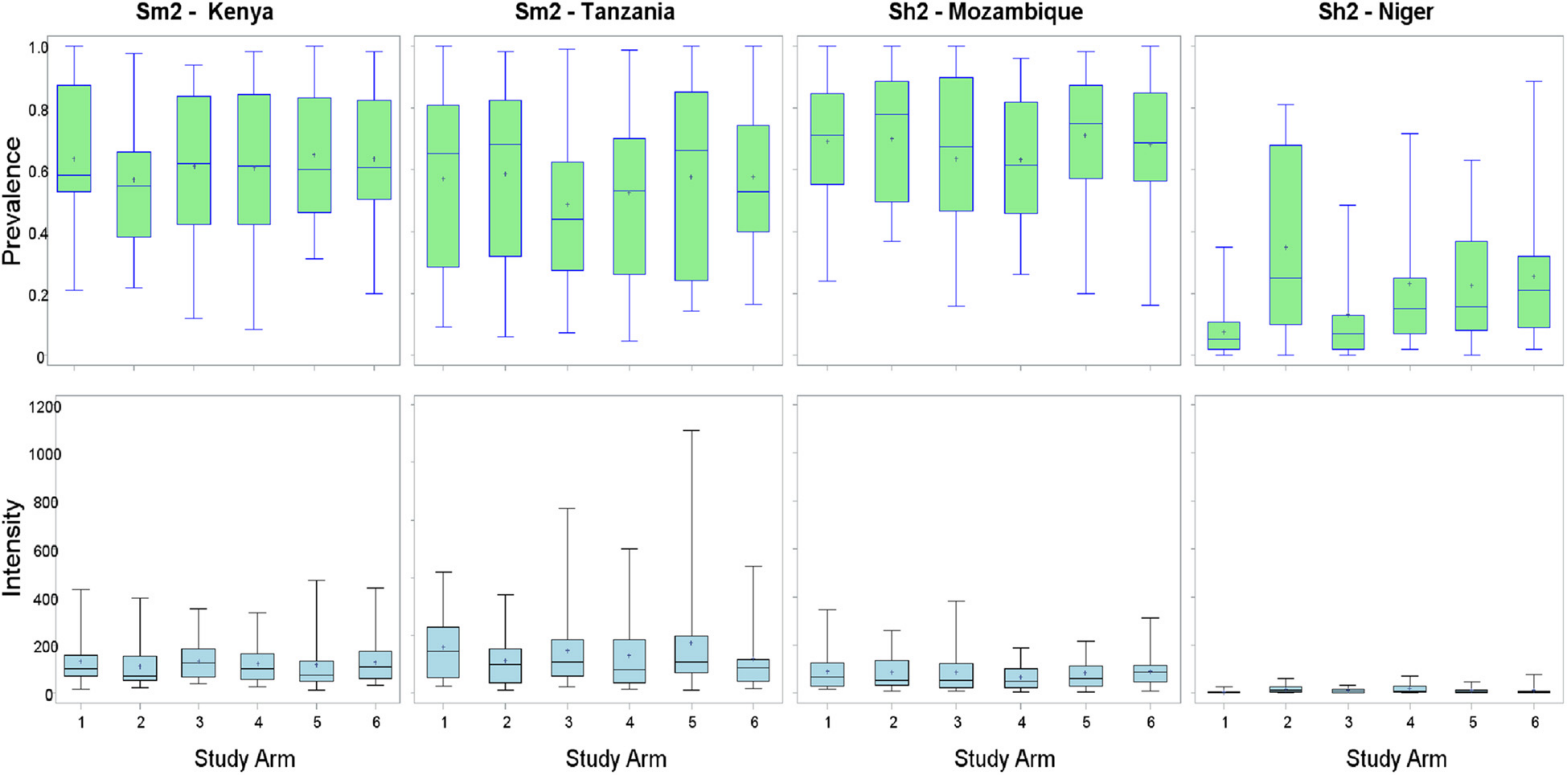
Baseline infection prevalence and intensity for gaining control of schistosomiasis studies, by study arm. Figures depict box plots. Horizontal lines in box interiors indicate medians. Box lengths represent the interquartile range (i.e. amount of data between the 75^th^ and 25^th^ percentile), + signs in boxes represent mean infection intensity (in eggs per gram of faeces (for *S. mansoni*) or per 10 ml of urine (for *S. haematobium*)) or prevalence in the respective arms 1–6 and the whiskers represent the minimum and maximum infection prevalence or intensity. Sh2, gaining control study in *S. haematobium* villages; Sm2, gaining control study in *S. mansoni* endemic villages

Studies of gaining control (Fig. 1b) in *S. mansoni* areas bordering Lake Victoria in the Kisumu region of western Kenya and Mwanza region of Tanzania included 11,541 and 14,620 children aged 9–12 years, respectively (Table 4). The prevalence of *S. mansoni* infection among children aged 9–12 years was 62.7 % in Kenya and 55.5 % in Tanzania. Among children aged 9–12 years, 7.3 % of villages in Kenya and 20.3 % of villages in Tanzania had a prevalence less than 25 %, despite prevalence ≥25 % in the eligibility survey. Numbers of children between 9 and 12 year of age enrolled per village ranged from 12 to 101 in Kenya, and from 11 to 122 in Tanzania. There were no significant differences in prevalence of infection or intensity among 9- to 12-year-old children by study arm in either country (Fig. 2).

**Table 4:** Numbers of participants and prevalence in the year 1 survey, by study type and country

Age group	Variable	Study - country						
		Sm2 - Kenya	Sm2 - Tanzania	Sh2 – Mozambique	Sh2 – Niger	Sm1 - Côte d’Ivoire	Sm1 - Kenya	Sh1 - Niger
5–8 years	Number enrolled	4,725	12,359	7,463	13,553	4,812	1,609	6,667
	Prevalence (%)	35.7	38.5	63.1	24.0	5.3	5.8	3.3
9–12 years	Number enrolled	11,541	14,620	7,317	14,249	7,410	4,614	6,682
	Prevalence (%)	62.7	55.5	66.6	21.3	20.9	17.7	4.2
Adults (20–55 years)	Number enrolled	7,107	4,922	4,259	7,041	N/A	N/A	N/A
	Prevalence (%)	44.7	28.1	44.8	11.3	N/A	N/A	N/A
Total	Number enrolled	23,373	31,901	19,039	34,843	12,222	6,223	13,349

*N/A* not assessed, *Sh1* sustaining control study in *S. haematobium* endemic villages, *Sh2* gaining control study in *S. haematobium* endemic villages, *Sm1* sustaining control study in *S. mansoni* endemic villages, *Sm2* gaining control study in *S. mansoni* endemic villages

Studies of gaining control of *S. haematobium* in Cabo Delgado province of northern Mozambique and the Dosso and Tillaberi regions of western Niger enrolled 7317 and 14,249 children aged 9–12 years, respectively (Table 4). The prevalence of *S. haematobium* infection was 66.6 % in Mozambique and 21.3 % in Niger. Approximately 5.3 % of villages in Mozambique and 67.3 % in Niger had prevalence less than 25 % in this age group, despite having had a prevalence ≥25 % among 13–14 year olds in the eligibility survey. The number of 9- to 12-year-old children enrolled per village ranged from 10 to 139 and from 51 to 149 in Mozambique and Niger, respectively. Both the prevalence and intensity of infection were similar among study arms (Fig. 2) in Mozambique. However, the *S. haematobium* infection prevalence was significantly different by study arms in Niger (*p* <0.001) (Fig. 2). Infection prevalence was lower among children aged 5–8 years and among adults, compared to children aged 9–12 years, in Kenya, Tanzania, Mozambique and Niger. However, in Niger Sh2 (Table 4), infection prevalence was slightly higher among younger children (24.0 %) than among those aged 9–12 years (21.3 %).

#### Studies of sustaining control (Côte d’Ivoire, Kenya and Niger)

**Fig. 3:**
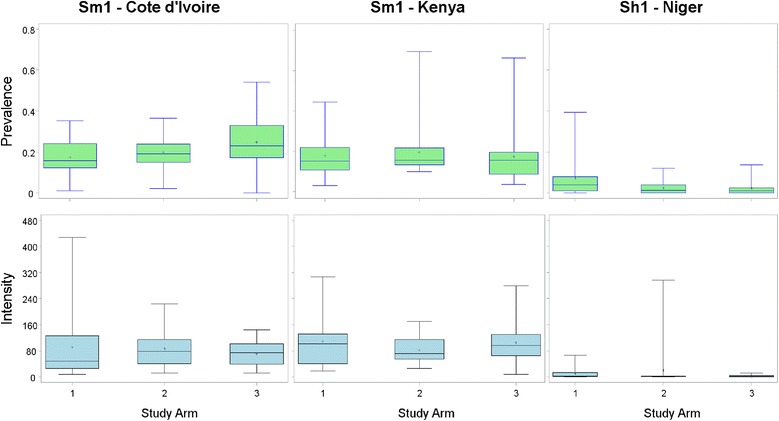
Infection prevalence and intensity for sustaining control of schistosomiasis studies, by study arm. Figures depict box plots. Horizontal lines in box interiors indicate medians. Box lengths represent the interquartile range (i.e. amount of data between the 75^th^ and 25^th^ percentile), + signs in boxes represent mean infection intensity (eggs per gram of faeces (for *S. mansoni*) or eggs per 10 ml of urine (for *S. haematobium*)) or prevalence in the respective arms 1–3 and the whiskers represent the minimum and maximum infection prevalence or intensity. Sh1, sustaining study in *S. haematobium* endemic villages; Sm1, sustaining study in *S. mansoni* endemic village

Studies of sustaining control in *S. mansoni* areas (Fig. 1b) included 7410 children aged 9–12 from Région des Montagnes and Région du Moyen Cavally in western Côte d’Ivoire and 4614 from Kenya (Table 4). The prevalence among these children was 20.9 % in Côte d’Ivoire and 17.7 % in western Kenya, near Lake Victoria, in an area distinct from that where the gaining control study was being conducted. In the baseline data on 9- to 12-year-old children, 43 % (*n* = 32) of villages in Côte d’Ivoire and 39 % (*n* = 29) in Kenya had year 1 prevalence outside the desired range of 10–24 %. Nine (12 %) Ivorian and 14 (19 %) Kenyan villages had infection prevalence <10 % and 23 (31 %) Ivorian and 15 (20 %) Kenyan villages had infection prevalence ≥25 %. The number of children aged 9–12 years enrolled per village ranged from 77 to 115 in Côte d’Ivoire and from 26 to 123 in Kenya. There were no significant differences in prevalence or intensity of *S. mansoni* infection by study arm in either country (Fig. 3). Adults were not recruited into studies of sustaining control.

Sustaining control studies of *S. haematobium* in Niger enrolled 6682 children aged 9–12 years from the Dosso and Tillaberi regions of western Niger, with a prevalence of infection of 4.2 % (Table 4). About 92 % of villages in Niger had year 1 prevalence outside the desired range of 10–24 %, mostly less than 10 %. The number of children enrolled per village ranged from 24 to 102 with 46 (61 %) of villages achieving the protocol enrollment target of 100. There were no significant differences in prevalence and intensity of *S. haematobium* infection by study arm in Niger (Fig. 3) (*p* = 0.122 and *p* = 0.111, respectively). Prevalence of infection among children aged 5–8 years was 3.3 % (Table 4).

## Discussion

The SCORE gaining and sustaining control studies are large operational intervention studies, involving complex coordination among multiple investigators from many countries, between research projects and national schistosomiasis control programmes (often integrated within a broader NTD master plan), and among national and subnational governmental levels and in-country community-based and non-governmental organisations. In year 1 alone, the project enrolled 66,433 children aged 9–12 years across 825 villages in five countries, in addition to large numbers of younger children (5–8 years) and adults (20–55 years). Despite these large numbers, enrolment in the year 1 study was somewhat lower than the expected 82,500. Possible reasons for this discrepancy include low school enrolment, lack of parental consent, limited pre-study sensitization in some areas, and flooding, political unrest and other contextual issues extraneous to the study. In addition to the logistical challenges of visiting 75 or 150 villages in each study, consenting and enrolling large numbers of people, and the demands of laboratory testing were considerable. The gaining and sustaining control surveys of *S. mansoni* were particularly difficult, requiring duplicate assessment of three consecutive stool specimens from children in the 9–12 year age group, as well as duplicate testing of a single stool specimen in young children and adults. For example, in the first year alone 214,122 Kato-Katz thick smears were read for the 38,185 enrolled children aged 9–12 years. Technicians read an additional 77,351 Kato-Katz thick smears from the 35,555 enrolled young children and adults. By design, the number of stools used for infection assessment differed by age-group and may in part explain the difference in the prevalence of infection between age-groups as previously reported [31].

The SCORE gaining and sustaining schistosomiasis control project made a dedicated effort as part of study planning activities to identify eligible villages with sufficient numbers of children for the respective studies. In all countries but Mozambique, many more villages had to be visited than anticipated to identify the number of potentially eligible villages for this study. This suggests that the historical data used to identify study areas was outdated or unreliable, which might be partially explained by escalating control efforts [32]. Despite eligibility survey prevalence in 13- to 14-year-old children that met study criteria (range: 10–24 %), on testing in year 1 of the actual study, 57.8 % of villages of eligible villages in the sustaining studies had prevalence below 10 % or above 24 % among children aged 9–12 years. In the gaining control studies, 26 % of villages with prevalence ≥25 % in the eligibility survey had prevalence levels <25 % in 9- to 12-year-old children in year 1 of the study. Possible explanations for the discordance between the eligibility and the year 1 survey results include that (i) the prevalence of *Schistosoma* infection in children aged 13–14 years was substantially higher than that among 9- to 12-year-old children in the same communities; (ii) differences in sampling methodology between eligibility and study sample stool assessments; and (iii) possible random inclusion of atyptical sample of 13- to 14-year-old children with high infection burden in the eligibility survey but infection level among children in the study sample were more typical in the 9- to 12-year-old children leading to regression of average infection levels towards the mean.

The harmonized protocol for gaining and sustaining control of schistosomiasis was successfully implemented in four of the five funded countries. Niger did not allocate interventions to villages as required by the protocol, but instead grouped study villages by their proximity to one another – three groups in the sustaining study and six in the gaining study. These geographically clustered groups were then randomly assigned to study arms. The differences in prevalence and intensity in the arms of the gaining study is a result of this failure to properly randomise at the village level.

**Fig. 4:**
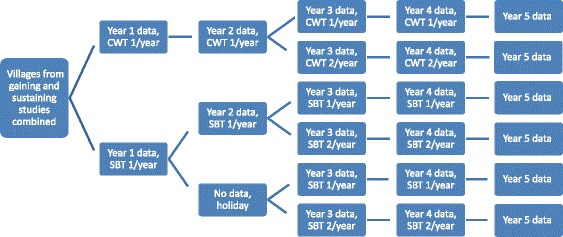
Revised study design for Niger. CWT, community-wide treatment; SBT, school-based treatment

Because the data being collected by the Niger team could not be used to answer the overarching gaining and sustaining questions, new research questions and a new study protocol were devised three years into the investigation, to compare the benefits of twice a year PCT compared with once a year (Fig. 4). In year 3, villages within each of the original study arms were randomly assigned to receive PZQ treatment either once or twice a year in an effort to make use of the two years of data collection and intervention that had already occurred.

Harmonization of data collection and management efforts across study teams and regions proved a formidable challenge. Although SCORE provided a list of variables to be collected and put forth specific forms, for example, for village inventory data, these were not universally followed. SCORE had planned to use mobile data collection and centralised data storage to ensure consistency; however, this was more difficult than anticipated. The expected adaptation of the software and use of the same mobile data collection tools used in lymphatic filariasis studies was not possible due to the extensive reprogramming required to adapt the software and to the evolving and rapid changes in the tools used for mobile data collection. SCORE partnered with Imperial College London to develop an application of their EpiCollect® software, but this took longer than expected and was not ready to be implemented by all sites in time for year 1 data collection. Since then, use of mobile devices for NTD programme data collection has expanded and advanced, and the ease of developing applications has increased markedly. Mobile systems (either EpiCollect or the LINKS system) are currently being used by four of the five study countries. Systems for data cleaning and submission to SCORE have been standardised, with data cleaned in-country, then submitted to SCORE, where they are reviewed and put into a standardised format (i.e. SCORE uniform data set (SUDS)). The uniform data sets are then returned to the originating country PIs, and can also be combined for multi-country analyses, as in the current paper.

We anticipate that the optimal PCT strategy for a given region may, in part, depend on the starting prevalence and intensity of infection and contextual local factors that must be carefully considered in the adoption for schistosomiasis control. The results of these gaining and sustaining studies will provide data-driven decision frameworks for national NTD control programme managers, as well as what should be an invaluable source for researchers and mathematical modellers. The infrastructure from this research laid by the SCORE programme has already contributed to important spin-off efforts, including schistosome infection modelling [33–36]. Of note, SCORE-related efforts or support have already resulted in more than 40 peer-reviewed publications and one non peer-reviewed white paper (all publications and the white paper can be accessed via the SCORE website, available on: http://www.score.uga.edu).

## Conclusion

World Health Assembly (WHA) resolution number 65.21 of March 2012 encourages the world to move towards elimination of schistosomiasis. Although the gaining and sustaining studies described here mainly focus on morbidity control by carefully examining different PCT schemes with PZQ, it is widely agreed that to achieve the reductions in prevalence and intensity needed to approach the goal of breaking transmission (i.e. elimination), enhanced PCT such as more frequent dosing than once a year, additional interventions, such as snail control, improvement in water, sanitation and hygiene (WASH), setting-specific information, education and communication (IEC), paediatric PZQ formulations and, possibly, drugs that more effectively kill adult worms and treat juvenile worms, are necessary [8, 37–44]. The results of these gaining and sustaining studies, however, will provide strategic information about how best to implement PCT and use treatment-related resources in countries with moderate and high prevalence of infection at the onset of multi-year treatment interventions.

## Abbreviations

CDC, centers for disease control and prevention; CWT, community-wide treatment; DALY, disability-adjusted life year; IEC, information, education and communication; IRB, institutional review board; KEMRI, Kenyan Medical Research Institute; NIMR, National Institute for medical research; NTD, neglected tropical disease; PCT, preventive chemotherapy; PI, principal investigator; PZQ, Praziquantel; RAP, rapid answers project; SAC, school-aged children; SBT, school-based treatment; SCI, schistosomiasis control initiative; SCORE, Schistosomiasis Consortium for Operational Research and Evaluation; Sh1, *Schistosoma haematobium* sustaining control study; Sh2, *Schistosoma haematobium* gaining control study; Sm1, *Schistosoma mansoni* sustaining control study; Sm2, *Schistosoma mansoni* gaining control study; SOP, standard operating procedure; SUDS, SCORE uniform data set; UGA, University of Georgia; WASH, water, sanitation and hygiene; WHA, World Health Assembly; WHO, World Health Organization
